# Cotton Fabric-Reinforced Hydrogels with Excellent Mechanical and Broad-Spectrum Photothermal Antibacterial Properties

**DOI:** 10.3390/polym16101346

**Published:** 2024-05-09

**Authors:** Xiangnan Yuan, Jun Zhang, Jiayin Shi, Wenfu Liu, Andreii S. Kritchenkov, Sandra Van Vlierberghe, Lu Wang, Wanjun Liu, Jing Gao

**Affiliations:** 1 Key Laboratory of Textile Science & Technology, Ministry of Education, College of Textiles, Donghua University, Shanghai 201620, China; 2 Engineering Research Center of Technical Textiles, Ministry of Education, College of Textiles, Donghua University, Shanghai 201620, China; 3 Shanghai Engineering Research Center of Nano-Biomaterials and Regenerative Medicine, Donghua University, Shanghai 201620, China; 4 College of Energy Engineering, Huanghuai University, Zhumadian 463000, China; 5 Institute of Environmental Engineering, Department of Human Ecology and Bioelementology, Peoples’ Friendship University of Russia (RUDN University), Moscow 117198, Russia; 6 Institute of Technical Acoustics NAS of Belarus, 210009 Vitebsk, Belarus; 7 Polymer Chemistry and Biomaterials Group, Centre of Macromolecular Chemistry, Department of Organic and Macromolecular Chemistry, Ghent University, 9000 Ghent, Belgium

**Keywords:** cotton fabric, reinforced hydrogel, photothermal therapy, mechanical properties, antibacterial properties

## Abstract

Antibacterial hydrogel wound dressings hold great potential in eliminating bacteria and accelerating the healing process. However, it remains a challenge to fabricate hydrogel wound dressings that simultaneously exhibit excellent mechanical and photothermal antibacterial properties. Here we report the development of polydopamine-functionalized graphene oxide (rGO@PDA)/calcium alginate (CA)/Polypyrrole (PPy) cotton fabric-reinforced hydrogels (abbreviated as rGO@PDA/CA/PPy FHs) for tackling bacterial infections. The mechanical properties of hydrogels were greatly enhanced by cotton fabric reinforcement and an interpenetrating structure, while excellent broad-spectrum photothermal antibacterial properties based on the photothermal effect were obtained by incorporating PPy and rGO@PDA. Results indicated that rGO@PDA/CA/PPy FHs exhibited superior tensile strength in both the warp (289 ± 62.1 N) and weft directions (142 ± 23.0 N), similarly to cotton fabric. By incorporating PPy and rGO@PDA, the swelling ratio was significantly decreased from 673.5% to 236.6%, while photothermal conversion performance was significantly enhanced with a temperature elevated to 45.0 °C. Due to the synergistic photothermal properties of rGO@PDA and PPy, rGO@PDA/CA/PPy FHs exhibited excellent bacteria-eliminating efficiency for *S. aureus* (0.57%) and *E. coli* (3.58%) after exposure to NIR for 20 min. We believe that the design of fabric-reinforced hydrogels could serve as a guideline for developing hydrogel wound dressings with improved mechanical properties and broad-spectrum photothermal antibacterial properties for infected-wound treatment.

## 1. Introduction

Bacterial infection can reduce the healing process of damaged wounds, thereby hindering the restoration of skin tissue, impacting the quality of life, and even threatening lives. For more severe cases, bacterial-infected wounds may lead to sepsis and multiorgan failure, which has a staggering 20–40% mortality rate [1,2,3]. Notably, over USD 31 billion has been spent annually on chronic wound treatment and management, bringing about a substantial burden to the national healthcare system [4]. Hydrogel wound dressings hold great potential in eliminating bacteria and accelerating the healing process [5,6,7]. To tackle bacterial infections at the wound site and create a better pro-healing microenvironment, various antibacterial agents (i.e., curcumin [8], polypeptide nisin [9]) and antibiotics (i.e., penicillin, vancomycin, and gentamicin) were integrated into hydrogel wound dressings to achieve efficient antibacterial effects, which would inevitably lead to unpredictable side effects. In addition, drug activity cannot be ensured during the entire complex wound-healing process [10,11]. Furthermore, more than 70% of wound-infected bacteria clinically appear resistant to at least one antibiotic.

By incorporating photothermal therapy agents, hydrogel wound dressings can convert optical energy into local heating and cause physical damage to pathogens [12,13,14,15]. Completely different from conventional antimicrobial therapy, photothermal therapy can prevent bacteria from developing drug resistance and has gained mounting attention in the treatment of wound infections due to its broad-spectrum antibacterial properties, noninvasiveness, and remote controllability [16]. Among various photothermal materials, polydopamine-functionalized graphene oxide (rGO@PDA) exhibits excellent thermal stability, high photothermal conversion capability, and superior biocompatibility, which is suitable for photothermal antibacterial wound therapy [17,18,19,20]. For example, photothermally responsive antimicrobial gelatin methacryloyl hydrogels have been fabricated for the treatment of wound infections based on rGO@PDA [21].

One major challenge for hydrogel wound dressings is their poor mechanical properties [22,23], which cannot provide enough physical support and are even deformed or broken when applied to the wound site [24,25]. To overcome this practical restriction, researchers have focused on constructing interpenetrating hydrogel networks [26,27,28]. For example, Wenzheng Li and coworkers [29] synthesized the double-network polyvinyl alcohol-polypyrrole hydrogels by the freeze–thaw and in situ polymerization method, leading to more stable structures. Results showed that the double-network structure significantly improved hydrogel elongation (156.4%), much higher than that of pristine polyvinyl alcohol hydrogels (103.0%). In addition, fabrics have been used to reinforce hydrogels to enable excellent mechanical properties for various applications including oil–water separation, soft sensors, tissue scaffolds, and wound dressing [30,31,32,33]. For example, Ran Zhang and coworkers reported that fabric-reinforced poly (vinyl alcohol) composite hydrogels exhibited significant improvement in mechanical properties compared with cotton fabric and neat PVA gel [31]. Compared to pure hydrogels, reinforcement with cotton fabric is straightforward, and cost-effective, which can provide robust mechanical properties in the long term. It should be noted that alginate hydrogels have been widely used in wound management because of their remarkable liquid adsorption, hemostatic properties, and biosafety, which are beneficial for wound healing [24,25,34]. Nevertheless, their poor mechanical properties often lead to structural collapse when applied at wound sites, especially for long-term use, which makes it difficult to achieve the desired treatment efficacy. To this end, it remains a challenge to fabricate hydrogel wound dressings that simultaneously exhibit excellent mechanical and photothermal antibacterial properties.

In this work, we present polydopamine-functionalized graphene oxide (rGO@PDA)/calcium alginate (CA)/Polypyrrole (PPy) fabric-reinforced hydrogels (abbreviated as rGO@PDA/CA/PPy FHs) for tackling bacterial infections. Notably, alginate hydrogels were selected as the main materials of wound dressings to directly interact with wound skin due to their superior biocompatibility and the capability to retain wound exudate for promoting healing process. In addition, alginate hydrogels also played an important role in integrating other functional components into composite wound dressings. Moreover, cotton fabric was employed to improve the mechanical properties of wound dressings by reinforcement. In addition, the mechanical properties of alginate hydrogels were further enhanced by generating an interpenetrating hydrogel network with PPy. Furthermore, PPy and rGO@PDA were employed to enable hydrogel wound dressings with superior broad-spectrum photothermal antibacterial properties based on the photothermal effect. Results indicated that rGO@PDA/CA/PPy FHs exhibited excellent tensile strength in the warp (289 ± 62.1 N) and weft directions (142 ± 23.0 N), similarly to cotton fabric. By incorporating PPy and rGO@PDA, the swelling ratio was significantly decreased from 673.5% to 236.6%, while photothermal conversion performance was significantly enhanced with a temperature elevated to 45.0 °C. Due to the synergistic photothermal conversion properties of rGO@PDA and PPy, rGO@PDA/CA/PPy FHs exhibited excellent bacteria-eliminating efficiency for *S. aureus* (0.57%) and *E. coli* (3.58%) after exposure to NIR for 20 min. Our work may facilitate the development of cotton fabric-reinforced hydrogels for infected-wound management.

## 2. Experimental Section

### 2.1. Materials

Sodium alginate, calcium carbonate (CaCO_3_), pyrrole (PPy), and dopamine (DA) hydrochloride were supplied by Shanghai Yishi Chemical Technology Co., Ltd., Shanghai, China. Iron (III) chloride hexahydrate (FeCl_3_·6H_2_O) and hydrochloric acid (HCl, 36.0%~38.0%) were bought from Sinopharm Chemical Reagent Co., Ltd., Shanghai, China. Tris(hydroxymethyl) aminomethane (Tris) was purchased from Macklin Co., Ltd., Shanghai, China. graphene oxide (GO) was supplied by Suzhou Tanfeng Graphene Technology Co., Ltd., Suzhou, China. Glucono-δ-lactone was bought from Shanghai Titan Co., Ltd., Shanghai, China. Cotton fabric (10 cm × 10 cm-8p) was acquired from Zhende Medical Co., Ltd., Shaoxing, China. All materials and reagents were used without further purification.

### 2.2. Synthesis of rGO@PDA

The synthesis of rGO@PDA was carried out according to a previous report [20] with minor changes. Specifically, 100 mg GO and 50 mg DA were dissolved in 200 mL of 10 mM Tris-HCl (pH = 8.5). Under ice conditions, the obtained solution was dispersed in an ultrasonic machine for 10 min. Next, it was heated at 60 °C under magnetic stirring for 24 h. Finally, the mixture was filtered and washed with deionized water. The product (rGO@PDA) was collected after drying for further use.

### 2.3. Preparation of Cotton Fabric-Reinforced Hydrogels

CaCO_3_ was dissolved in sodium alginate solution (0.02 g/mL, 12 g) under ultrasonic mixing with CaCO_3_–sodium alginate mass ratio of 0.287. Then, rGO@PDA was added into the mixture solution at different rGO@PDA–sodium alginate mass ratios (i.e., 0, 0.02, 0.04, 0.08, 0.16). Next, cotton fabric (5 cm × 5 cm) was immersed in the solution and then transferred to 25 g glucono-δ-lactone solution with a mass concentration of 0.9620% for 24 h. Obtained hydrogels were then immersed in FeCl_3_·6H_2_O solution (0.1024 M, 100 mL) for 24 h. After being washed with deionized water several times, the treated samples were put into PPy solution (0.0512 M, 100 mL) in the ice bath. HCl (0.1 M) was used to adjust the pH of the solution to 6. After 6 h of reaction and deionized water washing, rGO@PDA/CA/PPy FHs were finally obtained. For CA FHs and rGO@PDA/CA FHs, the PPy polymerization was skipped. In addition, there was no rGO@PDA for CA FHs during the fabrication process.

### 2.4. General Characterizations

A scanning electron microscope (TM-3000 SEM, Hitachi Co., Ltd., Shanghai, China) was used to observe the morphology. Fourier transform infrared spectroscopy was examined by an FTIR spectrometer (Nicolet 6700, Thermo Fisher Scientific, Waltham, MA, USA), with a wavenumber range of 4000–400 cm^−1^. A thermogravimeter (CLARUS SQ8-STA8000, Shanghai PerkinElmer Co., Ltd., Shanghai, China) equipped with a highly sensitive analytical balance was used for thermogravimetric analysis (TGA). Each sample placed in a corundum crucible was heated up to 800 °C (heating rate 10 °C·min^−1^) in a dynamic atmosphere of nitrogen with a flow rate of 30 mL·min^−1^.

### 2.5. Evaluation of Mechanical Properties

Tensile tests were conducted on an electronic fabric strength tester (YG(B)026G-500, Darong Textile Instrument Co., Ltd., Wenzhou, China) to characterize the mechanical properties. Experimental conditions were as follows: temperature 20 ± 3 °C, relative humidity 65% ± 5%, sample size 10 mm × 50 mm, tensile speed 10 mm/min, and clamping distance 40 mm. An average value was taken from three repeated experiments for each group.

### 2.6. Evaluation of the Swelling Behavior

The swelling behavior was quantified after rehydration of the samples in deionized water by measuring the weight changes as a function of immersion time. Samples with different rGO@PDA mass ratios were compared.

After measuring dry weights, wet weights were determined at different time points (i.e., 1 h, 2 h, 4 h, 8 h, 12 h, 24 h, 48 h) by gentle blotting with filter papers to remove exceeding surface liquid. The swelling ratio was calculated according to Equation (1) [21]:

(1) $$Swelling\left(\%\right)=\left(\frac{W_{s}-W_{d}}{W_{d}}\right)\times 100$$

where $W_{d}$ and $W_{s}$ are the weights of the samples in the dry and the swollen states, respectively. For each sample group, the results were taken as the mean values of three replicate samples under each condition.

### 2.7. Evaluation of Photothermal Properties

The obtained samples (sample size 10 × 10 mm) were placed into plastic centrifuge tubes that contained 1 mL of deionized water and irradiated with an 808 nm NIR light at 0.75 W/cm^2^ with a distance of 10 cm using a NIR irradiation device (HW808AD1000-34F, Shenzhen Infrared Laser Technology Co., Ltd., Shenzhen, China) for 20 min. Pure deionized water was used as the control group. Temperature changes and infrared images were recorded using a photothermal imager (TG165, FLIR, Portland, ME, USA) at different time points (i.e., 1 min, 2 min, 4 min, 8 min, 12 min, 16 min, 20 min), and the curve was drawn according to the temperature change.

### 2.8. Evaluation of In Vitro Antibacterial Efficiency

Antibacterial activity against *Staphylococcus aureus* (*S. aureus*, ATCC 25923) and *Escherichia coli* (*E. coli*, ATCC 25922) was measured by the spread plate assay. Briefly, 10 µL of PBS buffers (pH = 7.4) containing 10^9^ CFU·mL^−1^ bacteria were inoculated on a series of the experimental groups, including cotton fabric, CA FHs, CA/PPy FHs, and rGO@PDA/CA/PPy FHs. Then samples were exposed to NIR irritation for 20 min with the same parameters as mentioned in Section 2.7. Afterward, bacteria eluants from experimental samples were diluted 10^4^ times, and then the bacterial suspension (10 µL) from each group was spread onto agar plates. The plates were incubated for 18 h at 37 °C. The number of colonies on the agar plates was recorded, and the bacterial survival rate was calculated. Results were taken from the mean values of three replicate samples.

## 3. Results and Discussion

### 3.1. Design and Fabrication of rGO@PDA/CA/PPy FHs

**Figure 1:**
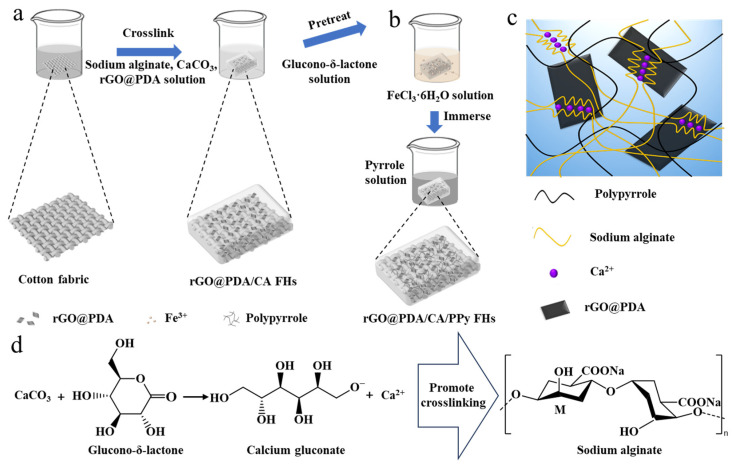
Design and fabrication of rGO@PDA/CA/PPy FHs. (**a**) Fabrication of rGO@PDA/CA FHs. (**b**) Fabrication of rGO@PDA/CA/PPy FHs. (**c**) The interpenetrating hydrogel network of rGO@PDA/CA/PPy FHs. (**d**) The promotion of the crosslinking process for alginate hydrogels based on the glucono-δ-lactone reaction system.

We developed rGO@PDA/CA/PPy FHs based on several considerations. Firstly, cotton fabric was used to reinforce the CA hydrogels and thus enhance mechanical properties. Secondly, rGO@PDA [35] was incorporated into the wound dressing to enable broad-spectrum antibacterial properties based on the photothermal effect (Figure 1a). Thirdly, in situ polymerization of pyrrole was employed to induce an interpenetrating hydrogel network for further improving the mechanical properties (Figure 1b,c). To fabricate cotton fabric-reinforced CA hydrogels, cotton fabric was soaked in sodium alginate, CaCO_3_, and rGO@PDA solution. To accelerate the crosslinking process, cotton fabric was transferred to a glucono-δ-lactone reaction system, which would react with CaCO_3_ and produce Ca^2+^, thereby forming an eggshell structure with sodium alginate for more efficient crosslinking progress (Figure 1d). In addition, the obtained fabric-reinforced hydrogels were treated by an oxidant agent of iron (III) chloride hexahydrate (Figure 1b), enabling the polymerization of PPy after being transferred to the PPy solution.

### 3.2. Structure and Mechanical Properties of Cotton Fabric-Reinforced CA Hydrogels

**Figure 2:**
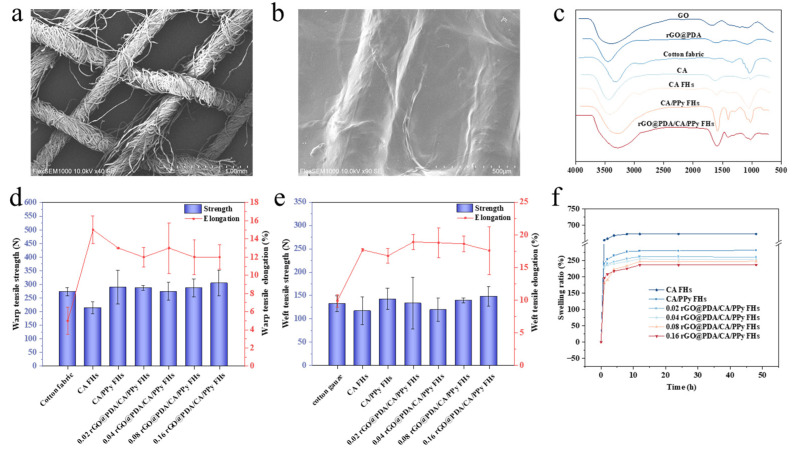
Structure and mechanical properties of cotton fabric-reinforced CA hydrogels. (**a**,**b**) SEM images of (**a**) cotton fabric and (**b**) rGO@PDA/CA/PPy FHs. (**c**) FTIR spectra of GO, rGO@PDA, cotton fabric, calcium alginate hydrogels, CA FHs, CA/PPy FHs, and rGO@PDA/CA/PPy FHs. (**d**) Warp breaking strength and breaking elongation of cotton fabric, CA/PPy FHs, and rGO@PDA/CA/PPy FHs with different rGO@PDA:SA mass ratios. (**e**) Weft breaking strength and breaking elongation of cotton fabric, CA/PPy FHs, and rGO@PDA/CA/PPy FHs with different rGO@PDA:SA mass ratios. (**f**) The swelling performance of CA FHs, CA/PPy FHs, and rGO@PDA/CA/PPy FHs with different rGO@PDA:SA mass ratios.

To confirm the feasibility of fabricating cotton fabric-reinforced CA hydrogels, SEM images were obtained (Figure 2a,b). Typical woven structures showing yarns and fibers were observed for the pristine cotton fabric (Figure 2a). After reinforcing CA hydrogels, both yarns and the gaps between yarns were covered by CA hydrogels, although the yarn shape still existed, indicating the successful fabrication of cotton fabric-reinforced CA hydrogels (Figure 2b). Notably, CA hydrogels were evenly attached to the fabric surface, which can ensure the structural stability of cotton fabric-reinforced hydrogels.

To validate the successful incorporation of desired functional components, FTIR spectra were obtained to characterize the functional groups (Figure 2c). Specifically, the FTIR spectrum of GO and rGO@PDA emerged at 1637 cm^−1^, which should be ascribed to the vibrations of C=C. In addition, the peak intensity at 1725 cm^−1^ (C=O) of rGO@PDA was lower than that of GO, indicating that the number of oxygen-containing groups of rGO@PDA decreased. Meanwhile, rGO@PDA exhibited a low intensity at 1456 cm^−1^ due to the bending vibration of N-H, demonstrating that GO has been successfully reduced by PDA. As for all FHs including calcium alginate FHs, calcium alginate/PPy FHs, and rGO@PDA/CA/PPy FHs, the -OH peak shifted to the lower frequency of 3419 cm^−1^ compared to that of cotton fabric (3443 cm^−1^). Compared to that of the pure cotton fabric, the peaks at 1629 cm^−1^ and 1400 cm^−1^ of all these FHs samples should be attributed to the asymmetrical -COO^−^ stretching vibration and symmetric -COO^−^ stretching vibrations of calcium alginate [24]. The characteristic peaks of PPy at 1030 cm^−1^ and 1317 cm^−1^ were present in calcium alginate/PPy FHs and rGO@PDA/CA/PPy FHs, verifying the successful deposition of PPy on FHs [25,27]. Taken together, the FTIR results verified that rGO@PDA/CA/PPy FHs were successfully fabricated.

To investigate whether the introduction of cotton fabric and the employment of the interpenetrating hydrogel network will improve mechanical performances, tensile tests were then conducted for cotton fabric, calcium alginate FHs, calcium alginate/PPy FHs and rGO@PDA/CA/PPy FHs with different rGO@PDA:SA mass ratios (Figure 2d,e). It should be noted that pure calcium alginate hydrogel broke very easily in the process of tensile experiments because of poor mechanical properties, so tensile experimental data could not be measured. In contrast, the warp and weft breaking strength of CA FHs were 213 ± 21.5 N, 117 ± 29.5 N, respectively, suggesting that reinforcement with cotton fabric can significantly improve the mechanical properties of CA hydrogels. Notably, breaking strength was further elevated in both warp (289 ± 62.1 N) and weft directions (142 ± 23.0 N) after polymerization of PPy, higher than that of cotton fabric (warp and weft breaking strength were 272 ± 15.3 N, 132 ± 16.8 N, respectively), which should be attributed to the existence of an interpenetrating structure. Specifically, the additional polymer networks of PPy in the alginate hydrogels provided the wound dressing with a more stable and compact structure against external force. Additionally, carboxyl groups of calcium alginate hydrogel could form a coordination structure with Fe^3+^ [28], which can further improve the overall toughness. It should be noted that no significant changes were detected after incorporating rGO@PDA. Nevertheless, our results indicated that the mechanical properties of hydrogels could be greatly improved by reinforcing with cotton fabric and employing the interpenetrating hydrogel network.

Besides tensile tests, swelling behaviors were also investigated to characterize the hydrogel stability (Figure 2f). It was obvious that all samples exhibited a faster swelling process at the early stage and then reached swelling equilibrium gradually. Inspiringly, the swelling ratio of calcium alginate/PPy FHs was 281.9% after 48 h, significantly lower than that of calcium alginate FHs, which reached 673.5% after 48 h, indicating that the introduction of PPy into the CA FHs can significantly reduce the swelling rate. The decreased swelling rate should be attributed to the existence of hydrophobic PPy in the CA FHs. In addition, the tight interpenetrating network caused by the crosslinking of the PPy molecular chain could also reduce the hydrogel water-binding capacity [26]. After adding rGO@PDA into calcium alginate/PPy FHs, the swelling ratio was further lowered in all rGO@PDA/CA/PPy FHs samples. In particular, 0.16rGO@PDA/CA/PPy FHs showed the lowest swelling ratio of 236.6% after 48 h, which should be ascribed to the fact that the presence of the layer structure of rGO@PDA could hinder the spatial extension of polymeric segments inside the hydrogel network. Furthermore, the hydrophobicity of the aromatic C=C of the graphene plane also affected the absorption capacity of the material [18,20]. To this end, the introduction of PPy and rGO@PDA can significantly improve the hydrogel stability by decreasing the swelling ratio. Remarkably, all rGO@PDA/CA/PPy FHs samples still retained a certain swelling capacity to ensure they absorb wound exudate in a timely fashion when serving as a wound dressing.

### 3.3. Thermal Properties of Cotton Fabric-Reinforced CA Hydrogels

**Figure 3:**
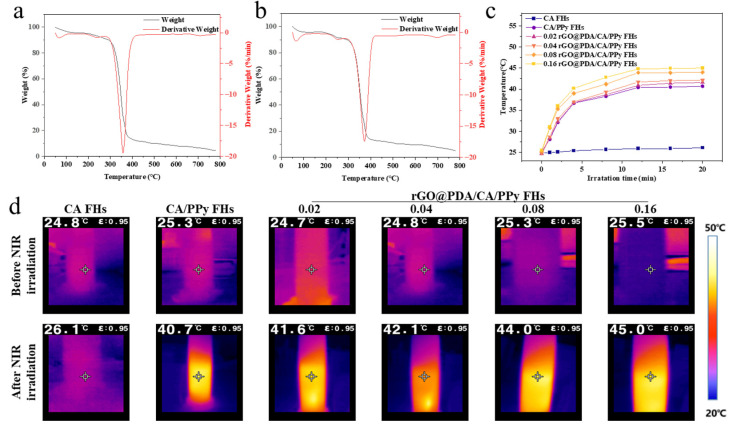
Thermal properties of cotton fabric-reinforced CA hydrogels. (**a**,**b**) Thermogravimetric curves and differential thermogravimetry curves of (**a**) CA/PPy FHs and (**b**) rGO@PDA/CA/PPy FHs. (**c**) The photothermal performance of CA FHs, CA/Ppy FHs, and rGO@PDA/CA/PPy FHs exposed to 808 nm NIR light laser for 20 min at 0.7 W cm^−2^: (**c**) photothermal heating curves and (**d**) the thermal images.

To verify the stability of cotton fabric-reinforced CA hydrogels for use in photothermal therapy, thermogravimetric curves were obtained for CA/PPy FHs and rGO@PDA/CA/PPy FHs (Figure 3a,b). To indicate the accumulation of weight loss, the differential thermogravimetry (DTG) curves, the first-order derivative of the corresponding TGA curves, were also obtained. For CA/PPy FHs, the weight loss occurred at about 294.8 °C, which should be assigned to the thermal decomposition of the organic groups and oxygen-containing functional groups of materials. After adding rGO@PDA, the initial decomposition temperature of samples was upshifted from 294.8 °C to 313.84 °C. According to the DTG curves, CA/PPy FHs showed a sharp peak at about 354.8 °C and a derivative weight of 19.6%/min, while rGO@PDA/CA/PPy FHs exhibited a strong peak at around 373.0 °C and a derivative weight of 18.1%/min, indicating that the addition of rGO@PDA shifted the decomposition temperature and increased the thermal stability. The above shifting should result from the fact that the proper dispersion of rGO@PDA in the composites enhanced interfacial interactions with the molecular chain, limiting the movement of the macromolecular chains. Meanwhile, the layered structure of rGO@PDA could also limit the small molecules generated during the pyrolysis process from leaving the system, leading to reduced mass loss [17,19]. Importantly, the weight loss below 100 °C belonged to the loss of water in the hydrogel, which was negligible, indicating that both CA/PPy FHs and rGO@PDA/CA/PPy FHs can meet the temperature requirement for the subsequent photothermal experiment.

To quantify photothermal conversion performance, the temperature was measured for CC/PPy FHs and rGO@PDA/CA/PPy FHs with different rGO@PDA:SA mass ratios exposed to 808 nm near-infrared light (NIR) laser for 20 min (Figure 3c,d). Notably, photothermal conversion performance was highly dependent on the existence of PPy and rGO@PDA. Specifically, the temperature of CA FHs showed negligible changes, while the temperature was enhanced from 26.1 °C to 40.7 °C after incorporating PPy. By increasing the rGO@PDA:SA ratio to 0.16, the temperature was further elevated to 45.0 °C. These changes demonstrate that rGO@PDA/CA/PPy FHs presented a synergistic photothermal effect from rGO@PDA and PPy. Specifically, rGO@PDA and PPy could exhibit strong optical absorption and then convert optical energy to thermal energy based on the energy-level transition principle, making it a promising photothermal wound dressing for antibacterial treatment.

### 3.4. Photothermal Antibacterial Properties of Cotton Fabric-Reinforced CA Hydrogels

**Figure 4:**
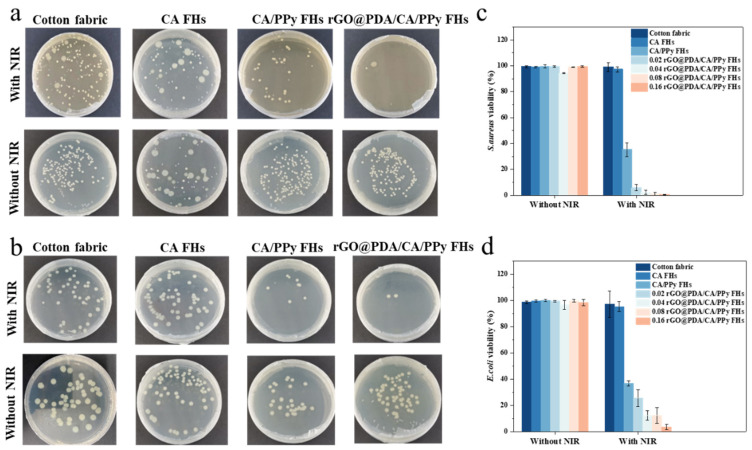
(**a**,**b**) Photographs of bacterial colonies formed by (**a**) *S. aureus* and (**b**) *E.coli* treated with various samples with or without 808 nm laser irradiation (0.7 W cm^−2^). (**c**,**d**) The bacterial viabilities of (**a**) *S. aureus* and (**b**) *E.coli* were measured using the plate count method.

Next, we further evaluated the *in vitro* photothermal antibacterial properties of cotton fabric-reinforced CA hydrogels against two bacterial strains (i.e., Gram-positive *S. aureus* and Gram-negative *E. coli*). After 18 h incubation, the colony formation units (CFU) of the viable bacteria were then counted (Figure 4). As expected, the survival rate of bacteria on cotton fabric and CA FHs showed no significant difference whether treated with NIR irradiation or not. Specifically, their *S. aureus* (Figure 4a,c) and *E. coli* (Figure 4b,d) bacterial survival rates were above 90%. Similarly, samples containing rGO@PDA or PPy did not show bacteria-killing capability when NIR irradiation was not involved. In contrast, the bacterial survival rate of CA/PPy FHs was 35.2% and 36.6% for *S. aureus* and *E. coli*, respectively, which should be attributed to the excellent photothermal conversion performance of PPy. Notably, rGO@PDA/CA FHs and rGO@PDA/CA/PPy FHs exhibited superior antibacterial effects regardless of bacterial type, which should be attributed to their effective heating for bacteria [14,15]. Notably, the rGO@PDA:SA mass ratio also showed a significant influence on the antibacterial properties. In particular, 0.16 rGO@PDA/CA/PPy FHs possessed the lowest bacterial survival rates of 0.57% and 3.58% for *S. aureus* and *E. coli*, respectively. These excellent photothermal antibacterial results were consistent with photothermal conversion results, suggesting that the mechanism of killing bacteria was attributed to direct thermal bacteria ablation. Specifically, rGO@PDA and PPy can convert optical energy into thermal energy upon NIR irradiation, resulting in high temperatures and then destroying the structural integrity of the bacterial cell wall and cell membrane. Meanwhile, the high temperature could also lead to protein enzyme denaturation, and even DNA damage, causing irreversible damage to bacteria [12,13,14].

To this end, we fabricated rGO@PDA/CA/PPy FHs that simultaneously exhibit excellent mechanical and broad-spectrum antibacterial properties enabled by NIR irradiation. All these properties are essential and required for materials when used as wound dressings. Our work has proven that rGO@PDA/CA/PPy FHs could achieve the required properties for practical wound management and serve as a robust physical barrier to avoid bacterial infection during the healing process. For standard clinical treatment with antibiotics (i.e., penicillin, vancomycin, and gentamicin), more than 70% of wound-infected bacteria clinically appear resistant to at least one antibiotic. In contrast, rGO@PDA/CA/PPy FHs with excellent broad-spectrum antibacterial properties can effectively kill both gram-positive and gram-negative bacteria with minimal side effects, providing an effective solution to addressing the clinical challenge of antibiotic resistance. We believe that the design of rGO@PDA/CA/PPy FHs could serve as a guideline for infected-wound clinical management. Notably, all materials including alginate [34], cotton fabric [9], rGO [36], PDA [37], and PPy [38] have been proven to exhibit superior biocompatibility in the literature. In addition, rGO nanomaterials were incorporated into the composite wound dressings, which can eliminate general safety concerns. Nevertheless, the current work focused on the antibacterial properties of rGO@PDA/CA/PPy FHs enabled by NIR irritation. Eliminating bacterial infection is just one major goal of wound dressings; functional components that promote wound healing are also needed to achieve desired wound management. Therefore, we believe that rGO@PDA/CA/PPy FHs hold great promise for medical use after further refining the wound-healing properties by combining other functional components.

## 4. Conclusions

In summary, we developed rGO@PDA/CA/PPy FHs with improved mechanical properties and photothermal antibacterial properties for wound management. Due to the cotton fabric reinforcement and the interpenetrating structure, rGO@PDA/CA/PPy FHs overcame the weakness of fragile calcium alginate hydrogels and exhibited excellent tensile strength in the warp (289 ± 62.1 N) and weft directions (142 ± 23.0 N), similarly to cotton fabric. By incorporating PPy and rGO@PDA, the swelling ratio was also significantly decreased from 673.5% to 236.6%, while photothermal conversion performance was significantly enhanced with a temperature elevated to 45.0 °C. Furthermore, the antibacterial experiments showed that rGO@PDA/CA/PPy FHs had excellent bacteria-eliminating efficiency for *S. aureus* (0.57%) and *E. coli* (3.58%) after exposure to NIR for 20 min due to the synergistic photothermal antibacterial properties of rGO@PDA and PPy. We believe that rGO@PDA/CA/PPy FHs hold great promise for medical use after further refining the wound-healing properties by combining other functional components. In addition, the design of fabric-reinforced hydrogels could serve as a guideline for developing hydrogel wound dressings with improved mechanical properties and broad-spectrum photothermal antibacterial properties for infected-wound treatment.

## Data Availability

The original contributions presented in the study are included in the article material, further inquiries can be directed to the corresponding authors.

## References

[1] Fleischmann-Struzek C., Mellhammar L., Rose N., Cassini A., Rudd K.E., Schlattmann P., Allegranzi B., Reinhart K. (2020). Incidence and mortality of hospital- and ICU-treated sepsis: Results from an updated and expanded systematic review and meta-analysis. Intensive Care Med..

[2] Bauer M., Gerlach H., Vogelmann T., Preissing F., Stiefel J., Adam D. (2020). Mortality in sepsis and septic shock in Europe, North America and Australia between 2009 and 2019—Results from a systematic review and meta-analysis. Crit. Care.

[3] Maitz J., Merlino J., Rizzo S., McKew G., Maitz P. (2023). Burn wound infections microbiome and novel approaches using therapeutic microorganisms in burn wound infection control. Adv. Drug Deliv. Rev..

[4] Li S., Renick P., Senkowsky J., Nair A., Tang L. (2020). Diagnostics for Wound Infections. Adv. Wound Care.

[5] Diacovich L., Gorvel J.-P. (2010). Bacterial manipulation of innate immunity to promote infection. Nat. Rev. Microbiol..

[6] Xu C., Akakuru O.U., Ma X., Zheng J., Zheng J., Wu A. (2020). Nanoparticle-Based Wound Dressing: Recent Progress in the Detection and Therapy of Bacterial Infections. Bioconjugate Chem..

[7] Shi L., Lin F., Zhou M., Li Y., Li W., Shan G., Xu Y., Xu J., Yang J. (2021). Preparation of biocompatible wound dressings with dual release of antibiotic and platelet-rich plasma for enhancing infected wound healing. J. Biomater. Appl..

[8] Sun X., Wu J., Wang H., Guan C. (2021). Thermosensitive Cotton Textile Loaded with Cyclodextrin-complexed Curcumin as a Wound Dressing. Fibers Polym..

[9] Gunes O.C., Ziylan Albayrak A. (2021). Antibacterial Polypeptide nisin containing cotton modified hydrogel composite wound dressings. Polym. Bull..

[10] Rosselle L., Cantelmo A.R., Barras A., Skandrani N., Pastore M., Aydin D., Chambre L., Sanyal R., Sanyal A., Boukherroub R. (2020). An ‘on-demand’ photothermal antibiotic release cryogel patch: Evaluation of efficacy on an ex vivo model for skin wound infection. Biomater. Sci..

[11] Zhang L., Wang Y., Wang J., Wang Y., Chen A., Wang C., Mo W., Li Y., Yuan Q., Zhang Y. (2019). Photon-Responsive Antibacterial Nanoplatform for Synergistic Photothermal-/Pharmaco-Therapy of Skin Infection. ACS Appl. Mater. Interfaces.

[12] Wang Y., Yang Y., Shi Y., Song H., Yu C. (2020). Antibiotic-Free Antibacterial Strategies Enabled by Nanomaterials: Progress and Perspectives. Adv. Mater..

[13] Chen Y., Gao Y., Chen Y., Liu L., Mo A., Peng Q. (2020). Nanomaterials-based photothermal therapy and its potentials in antibacterial treatment. J. Control. Release.

[14] Borzenkov M., Pallavicini P., Taglietti A., D’Alfonso L., Collini M., Chirico G. (2020). Photothermally active nanoparticles as a promising tool for eliminating bacteria and biofilms. Beilstein J. Nanotechnol..

[15] Bhattarai D.P., Tiwari A.P., Maharjan B., Tumurbaatar B., Park C.H., Kim C.S. (2019). Sacrificial template-based synthetic approach of polypyrrole hollow fibers for photothermal therapy. J. Colloid Interface Sci..

[16] Guan G., Win K.Y., Yao X., Yang W., Han M.-Y. (2021). Plasmonically Modulated Gold Nanostructures for Photothermal Ablation of Bacteria. Adv. Healthc. Mater..

[17] Peyravi A., Ahmadijokani F., Arjmand M., Hashisho Z. (2022). Graphene oxide enhances thermal stability and microwave absorption/regeneration of a porous polymer. J. Hazard. Mater..

[18] Francolini I., Perugini E., Silvestro I., Lopreiato M., Scotto d’Abusco A., Valentini F., Placidi E., Arciprete F., Martinelli A., Piozzi A. (2019). Graphene Oxide Oxygen Content Affects Physical and Biological Properties of Scaffolds Based on Chitosan/Graphene Oxide Conjugates. Materials.

[19] Sima H., Sui Y., Zhang C. (2022). Preparation of polysiloxane foam with graphene for promoting electromagnetic interference shielding performance and thermal stability. J. Appl. Polym. Sci..

[20] Gad Y.H., Nasef S.M. (2021). Radiation synthesis of graphene oxide/composite hydrogels and their ability for potential dye adsorption from wastewater. J. Appl. Polym. Sci..

[21] Cheng F., Xu L., Zhang X., He J., Huang Y., Li H. (2024). Generation of a photothermally responsive antimicrobial, bioadhesive gelatin methacryloyl (GelMA) based hydrogel through 3D printing for infectious wound healing. Int. J. Biol. Macromol..

[22] Chatterjee S., Mahmood S., Hilles A.R., Thomas S., Roy S., Provaznik V., Romero E.L., Ghosal K. (2023). Cationic starch: A functionalized polysaccharide based polymer for advancement of drug delivery and health care system—A review. Int J Biol Macromol.

[23] Rajakumari R., Saha P., Ghosal K., Tharayil A., Kalarikkal N., Thomas S., Jana S., Jana S., Domb A.J. (2022). Polysaccharide-based Scaffolds for Tissue Engineering Applications. Polysaccharide-based Biomaterials: Delivery of Therapeutics and Biomedical Applications.

[24] Xiang J., Shen L., Hong Y. (2020). Status and future scope of hydrogels in wound healing: Synthesis, materials and evaluation. Eur. Polym. J..

[25] Zhang M., Chen S., Zhong L., Wang B., Wang H., Hong F. (2020). Zn2+-loaded TOBC nanofiber-reinforced biomimetic calcium alginate hydrogel for antibacterial wound dressing. Int. J. Biol. Macromol..

[26] Xu X., Wang L., Jing J., Zhan J., Xu C., Xie W., Ye S., Zhao Y., Zhang C., Huang F. (2022). Conductive Collagen-Based Hydrogel Combined With Electrical Stimulation to Promote Neural Stem Cell Proliferation and Differentiation. Front. Bioeng. Biotechnol..

[27] Bu Y., Xu H., Li X., Xu W., Yin Y., Dai H., Wang X., Huang Z., Xu P. (2018). A conductive sodium alginate and carboxymethyl chitosan hydrogel doped with polypyrrole for peripheral nerve regeneration. RSC Adv..

[28] Anamizu M., Tabata Y. (2019). Design of injectable hydrogels of gelatin and alginate with ferric ions for cell transplantation. Acta Biomater..

[29] Li W., Chen W., Ma L., Yang J., Gao M., Wang K., Yu H., Lv R., Fu M. (2023). Robust double-network polyvinyl alcohol-polypyrrole hydrogels as high-performance electrodes for flexible supercapacitors. J. Colloid Interface Sci..

[30] Ye D., Cheng Q., Zhang Q., Wang Y., Chang C., Li L., Peng H., Zhang L. (2017). Deformation Drives Alignment of Nanofibers in Framework for Inducing Anisotropic Cellulose Hydrogels with High Toughness. ACS Appl. Mater. Interfaces.

[31] Zhang R., Wu Y., Lin P., Jia Z., Zhang Y., Liu F., Yu B., Zhou F. (2020). Extremely Tough Hydrogels with Cotton Fibers Reinforced. Adv. Eng. Mater..

[32] Li B., Li D., Yang Y., Zhang L., Xu K., Wang J. (2018). Study of thermal-sensitive alginate-Ca2+/poly(N-isopropylacrylamide) hydrogels supported by cotton fabric for wound dressing applications. Text. Res. J..

[33] Liu H., Yang L., Dou B., Lan J., Shang J., Lin S. (2021). Zwitterionic hydrogel-coated cotton fabrics with underwater superoleophobic, self-healing and anti-fouling performances for oil-water separation. Sep. Purif. Technol..

[34] Estes Bright L.M., Griffin L., Mondal A., Hopkins S., Ozkan E., Handa H. (2022). Biomimetic gasotransmitter-releasing alginate beads for biocompatible antimicrobial therapy. J. Colloid Interface Sci..

[35] Robinson J.T., Tabakman S.M., Liang Y., Wang H., Sanchez Casalongue H., Vinh D., Dai H. (2011). Ultrasmall Reduced Graphene Oxide with High Near-Infrared Absorbance for Photothermal Therapy. J. Am. Chem. Soc..

[36] Cao L., Lu C., Wang Q., Li F. (2017). Biocompatibility and fabrication of RGO/chitosan film for cartilage tissue recovery. Environ. Toxicol. Pharmacol..

[37] Panda S., Jeong H., Hajra S., Rajaitha P.M., Hong S., Kim H.J. (2023). Biocompatible polydopamine based triboelectric nanogenerator for humidity sensing. Sens. Actuators B.

[38] Cui S., Mao J., Rouabhia M., Elkoun S., Zhang Z. (2021). A biocompatible polypyrrole membrane for biomedical applications. RSC Adv..

